# Nano-Silica-Modified Concrete: A Bibliographic Analysis and Comprehensive Review of Material Properties

**DOI:** 10.3390/nano12121989

**Published:** 2022-06-09

**Authors:** Kaffayatullah Khan, Waqas Ahmad, Muhammad Nasir Amin, Sohaib Nazar

**Affiliations:** 1 Department of Civil and Environmental Engineering, College of Engineering, King Faisal University, Al-Ahsa 31982, Saudi Arabia; mgadir@kfu.edu.sa; 2 Department of Civil Engineering, COMSATS University Islamabad, Abbottabad 22060, Pakistan; waqasahmad@cuiatd.edu.pk (W.A.); sohaibnazar@cuiatd.edu.pk (S.N.)

**Keywords:** nanomaterials, nano-silica, cementitious materials, mechanical properties, durability, microstructure, scientometric analysis

## Abstract

Several review studies have been performed on nano-silica-modified concrete, but this study adopted a new method based on scientometric analysis for the keywords’ assessment in the current research area. A scientometric analysis can deal with vast bibliometric data using a software tool to evaluate the diverse features of the literature. Typical review studies are limited in their ability to comprehensively and accurately link divergent areas of the literature. Based on the analysis of keywords, this study highlighted and described the most significant segments in the research of nano-silica-modified concrete. The challenges associated with using nano-silica were identified, and future research is directed. Moreover, prediction models were developed using data from the literature for the strength estimation of nano-silica-modified concrete. It was noted that the application of nano-silica in cement-based composites is beneficial when used up to an optimal dosage of 2–3% due to high pozzolanic reactivity and a filler effect, whereas a higher dosage of nano-silica has a detrimental influence due to the increased porosity and microcracking caused by the agglomeration of nano-silica particles. The mechanical strength might enhance by 20–25% when NS is incorporated in the optimal amount. The prediction models developed for predicting the strength of nano-silica-modified concrete exhibited good agreement with experimental data due to lower error values. This type of analysis may be used to estimate the essential properties of a material, therefore saving time and money on experimental tests. It is recommended to investigate cost-effective methods for the dispersion of nano-silica in higher concentrations in cement mixes; further in-depth studies are required to develop more accurate prediction models to predict nano-silica-modified concrete properties.

## 1. Introduction

Utilizing resources economically and effectively has become a priority nowadays. Cement, being the primary component of concrete, has been under criticism in recent decades for the amount of CO_2_ emitted during its manufacture [1,2,3]. While cement possesses superior mechanical characteristics, it has been unable to meet the standards of durability [4,5,6,7]. Due to the limitations of cement, researchers began exploring additives that may improve the concrete properties while simultaneously making it lighter and more durable [8,9,10,11,12]. To increase the compactness, strength, and durability of cementitious materials, fly ash, silica fume, and other microparticles were used [13,14,15,16]. Additionally, these additives were chosen since they are industrial waste products that may be used responsibly and are also eco-friendly [17,18,19,20]. Fly ash is not favorable for initial strength gain and setting time and, hence, has certain drawbacks [21]. Silica fume, in combination with ceramic waste and rice husk ash, has lately attracted researchers’ interest, owing to its impact on the performance of cementitious materials [22]. Rice husk ash is a by-product of rice production that might be more efficiently exploited by substituting for 10–15% of cement [23,24,25]. The durability performance of rice husk ash concrete and recycled ceramic waste concrete has been improved in studies [26,27]. The production procedure for RHA is quite laborious, which raises concerns.

**Figure 1:**
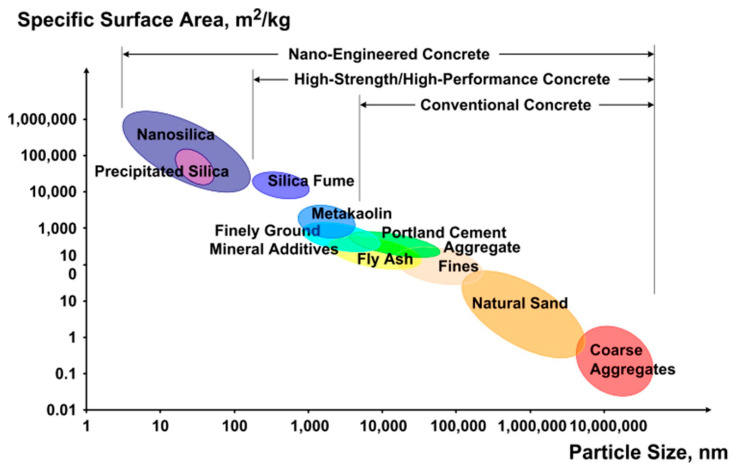
Particle size and SSA related to cementitious materials [58].

Researchers have achieved new heights in the realm of nanotechnology with the discovery of nano-particles (NP) finer than 100 nm in size [28,29,30]. NP may be used to improve the mechanical characteristics of various materials, such as polymers [31,32,33] and cementitious materials [34,35,36,37], and are also useful in the medical, engineering, and food domains [38,39,40,41]. This prompted researchers to perform more research on the impact of nano-silica (NS) on concrete [42]. Numerous NP have been investigated, including nano ZnO, nano Fe_2_O_3_, nano Al_2_O_3_, nano TiO_2_, and NS. Among all these NP, the utilization of NS in concrete enhanced compressive strength (CS) the most [43]. Additionally, NS shortened the initial and final setting times of the concrete and increased its early age strength. The most important component of NS is its nanostructure, which provides an unusually large specific surface area (SSA) and, hence, performs as an aggregate–cement binder [44]. NS’s significant pozzolanic activity is ascribed to its nano-particle size [45,46]. The interfacial transition zone (ITZ), which is regarded as a weak phase in cementitious materials, is also improved [47] since these NP pack in all gaps and voids due to their tiny size [48], hence decreasing permeability. NS has been shown to be a highly active component that accelerates the hydration process of cementitious materials [49] and forms more calcium-silicate-hydrate (C-S-H) gel [50,51], which is liable for the material strength [52,53]. The proportion of portlandite-Ca(OH)_2_ in cementitious materials decreases when NS combines with Ca(OH)_2_ to generate a denser product [54]. Some previous studies have indicated that substituting NS for up to 4% of the cement in concrete can enhance its mechanical strength and durability under adverse circumstances such as elevated temperatures and corrosion [55,56]. Though various researchers have confirmed the use of NS for particular applications of cementitious materials, it has been shown to be highly successful when utilized in a 0.5 to 4% proportion as a cement substitution. The excess amount of NS may cause agglomeration, owing to improper dispersion, hence limiting workability [57]. One of the most distinguishing characteristics of NP is their high volume-to-surface area ratio, as seen in Figure 1 [58]. Numerous NP are employed as nano additives in cementitious composites to improve their macroscopic characteristics and functioning; NS have become prevalent among those NP. However, the restricted practical uses of NS in construction are because of the high cost of NS, which is still 1000 times more expensive than ordinary cement [59,60].

Although studies on NS concrete progress in response to growing mechanical and durability issues, scientists are confronted with information limitations that may impede innovative exploration and academic collaboration. Consequently, it is crucial to develop and implement a system that enables researchers to acquire essential information from the most credible sources possible. Using a software program, a scientometric technique may help to overcome this deficiency. This study’s objective was to perform a keywords’ assessment utilizing scientometric analysis of bibliographic records published on NS-modified concrete up to 2021. A scientometric analysis can conduct a quantitative assessment of enormous bibliometric data by utilizing the appropriate software application. The capacity of conventional review studies to connect disparate areas of the literature in a comprehensive and accurate manner is limited. Science mapping, co-occurrence, and co-citation are among the most difficult aspects of contemporary exploration. Through the use of scientific maps, a scientometric study may find the most frequent and interconnected terms in a certain research subject. The Scopus database was used to extract bibliometric information from 1015 relevant papers, which were then analyzed using the VOSviewer application. Additionally, this study emphasized and outlined the most important areas of research in the field of NS-modified concrete. The difficulties inherent in the use of NS are highlighted, and future research is directed accordingly. Furthermore, this study performed a regression analysis of the literature data for establishing prediction models for the compressive, split-tensile, and flexural strengths of NS-modified concrete. This type of analysis might be used to estimate the desired properties of a material, hence reducing the expenses related to experimental works and saving time.

## 2. Review Strategy

For the assessment of keywords in the studies of NS-modified concrete, a scientometric analysis [61,62,63] of bibliographic data was conducted in this study. Numerous articles have been written on the subject; thus, it is essential to utilize a credible search engine. Scopus and Web of Science are two extremely precise search engines that are ideally suited for this purpose [64,65,66]. Scopus, which is highly recommended by academics [67,68], was used to collect the bibliographic material for this investigation on NS-modified concrete. A Scopus search for “nano-silica concrete” returned 1271 articles in April 2022. Numerous filter settings were utilized to eliminate unnecessary papers. These document kinds were chosen as journal article, journal review, conference paper, and conference review. The source types “journal” and “conference proceedings” were selected. The upper limit for “publishing year” was set to “2021”, while the language restriction was set to “English”. Following the implementation of these criteria, 1015 records were maintained. Researchers have also described a similar method in prior investigations [69,70].

**Figure 2:**
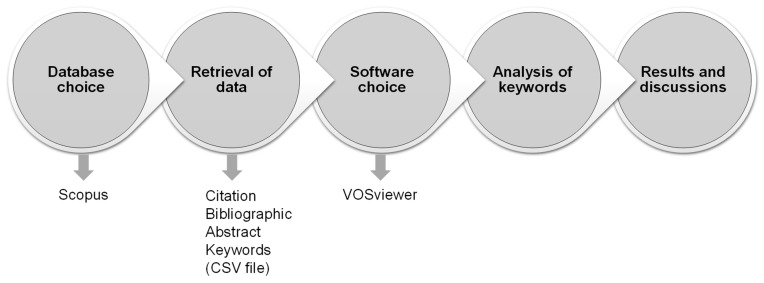
Sequence of the procedure followed for keywords’ analysis.

Scientometric studies utilize scientific maps, a technique established by academics for bibliometric data analysis [71]. Scopus records were stored as comma-separated value (CSV) files so that they could be evaluated using the relevant software. VOSviewer (version 1.6.17, Leiden University, Leiden, The Netherlands) was utilized to construct the scientific visualization and quantitative evaluation of the obtained data. VOSviewer is a readily accessible, open-source mapping application that is widely used in a variety of fields and recommended by academics [72,73,74,75,76]. Consequently, the current study’s objectives were met by the usage of VOSviewer. The resulting CSV files were imported to the VOSviewer, and further evaluation was conducted while maintaining data integrity and consistency. During the bibliographic evaluation, the most frequently occurring keywords were evaluated. The multiple features and their interrelationships and co-occurrence are illustrated graphically, and their statistical data are presented in a table. Figure 2 depicts the flowchart of the scientometric strategy. In addition, based on the analysis of keywords, the significant aspects of the present research were emphasized and described in detail. Additionally, regression analysis was performed in order to construct prediction models for the strength properties of NS-modified concrete.

## 3. Analysis of Results

**Figure 3:**
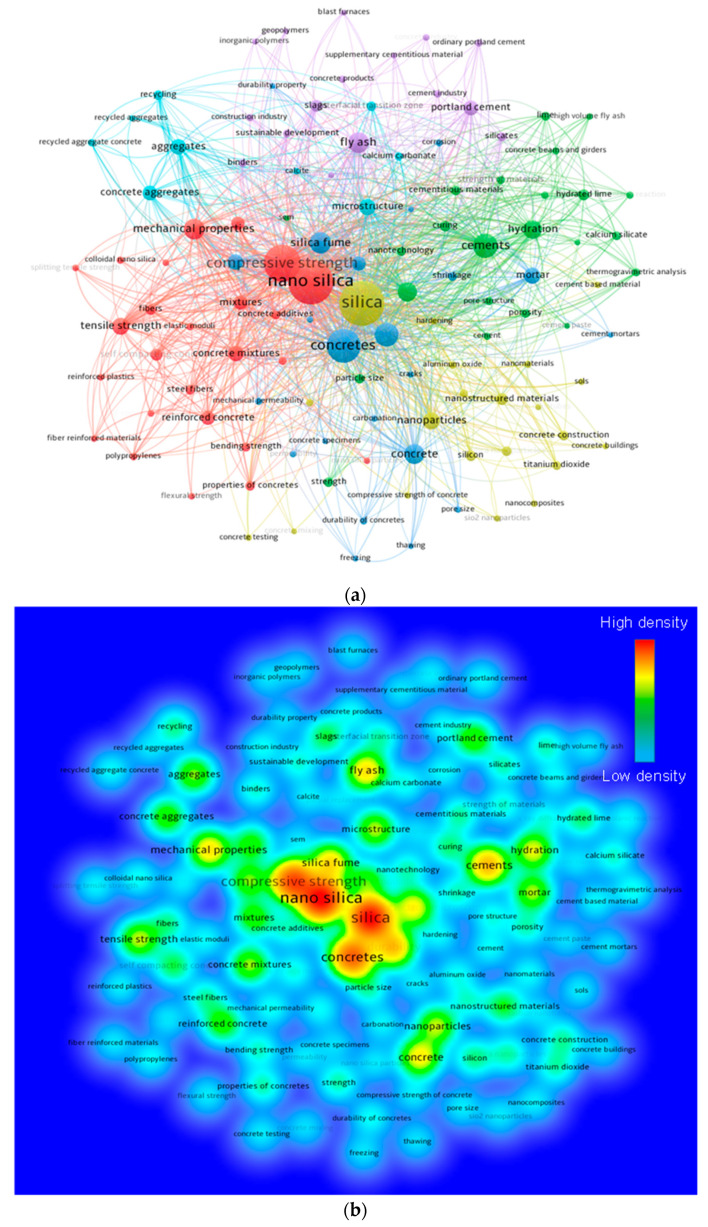
Keywords’ analysis: (**a**) scientific visualization; (**b**) density visualization.

Keywords are significant in research because they define and emphasize the core subject of the study domain [77]. For the assessment, the “analysis type” was set to “co-occurrence” and the “analysis unit” was set to “all keywords”. The minimum repetition requirement for a keyword was kept at 20, and 116 of the 5592 keywords were preserved. Table 1 lists the top 20 keywords most frequently used in published works on the subject. Nano-silica, silica, compressive strength, concretes, and cements are the five most often occurring terms in this field of study. According to the keyword analysis, NS has mostly been explored to enhance the durability, microstructural, and mechanical performances of concretes. Figure 3 illustrates a visualization map of keywords in terms of co-occurrences, connections, and frequency of occurrence density. The size of a keyword node in Figure 3a indicates its frequency, whereas its position indicates its co-occurrence in publications. In addition, the graph demonstrates that the top keywords have larger nodes than the rest, indicating that they are essential for NS concrete research. The graph highlights clusters in a manner that shows their co-occurrence in a variety of publications. The color-coded grouping was determined by the co-occurrence of several keywords in published works. Six separate hues (blue, red, green, purple, cyan, and yellow) represent the existence of six clusters (Figure 3a). As observed in Figure 3b, different colors represent differing keyword density concentrations. The hues red, yellow, green, and blue are arranged according to their density concentrations, with red representing the highest density concentration and blue representing the lowest. Silica, compressive strength, concretes, and nano-silica all display red indicators, indicating a greater density concentration. This discovery will help aspiring authors select keywords that accelerate the identification of published data in a certain topic.

## 4. Findings and Discussions

A scientometric assessment of the keywords in the field of NS concrete was conducted to determine the most frequently employed keywords and the linkage of keywords. After conducting a scientometric analysis of keywords on the bibliometric data, this study highlighted the most significant segments in the subject research area, which are covered briefly in the subsequent sub-sections. This determination was made after a thorough examination of all keywords and a study of the most pertinent literature. Previously, comparable approaches were adopted by other scholars in diverse research areas [78,79,80].

### 4.1. Properties of Nano-Silica

**Figure 4:**
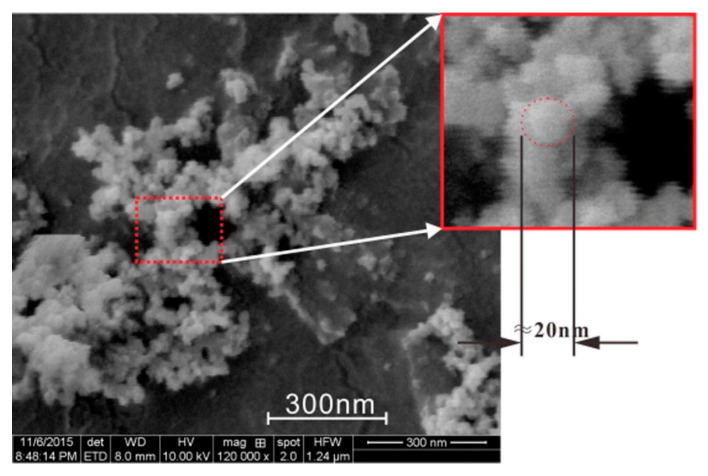
Micrograph of nano-silica [86].

The rapid reactivity of NS particles has received interest among diverse nanomaterials. It has accrued further advantages in the glass and concrete industries [81]. NS may be gel, precipitated, or pyrogenic [82]. Colloidal NS is composed of amorphous hydroxylated silica particles ranging in size from 1–500 nm in an aqueous solution [83]. The micrograph of NS is depicted in Figure 4. NS can be used to enhance the durability and mechanical properties of cementitious materials [84]. Substituting NS for cement to a certain amount can help cut CO_2_ emissions and make concrete more cost efficient [85]. NS combines with Ca(OH)_2_ in cementitious materials to generate C-S-H gel, which plugs the pores and, therefore, improves the early strength. Additionally, NS significantly lowers the porosity of cementitious materials compared to other traditional mineral admixtures due to its superior filling action and particle size distribution [85]. The superior SSA of the NS necessitates a high water–cement ratio (*w*/*c*) or a greater dose of super-plasticizers for improved flow characteristics and to minimize particle agglomeration. The distribution level of the NS used in the experiment has a substantial effect on the microstructure at the nanoscale. To have a deeper knowledge of the materials utilized in research, it is vital to investigate their physical and chemical characteristics. The physical features of NS are listed in Table 2. Table 3 summarizes the chemical compositions of several forms of NS.

### 4.2. Dispersion of Nano-Silica

Though NS is distributed effectively in water, it tends to aggregate in alkaline solutions due to the accessible Ca ions in the pore solution adsorbing on the surface of NS, hence initiating flocculation [98]. Although polycarboxylate super-plasticizers (PCE) are often employed to reduce the amount of water in a solution, they have been shown to be an effective dispersion for stabilizing nanomaterials such as nano clay particles [99] and graphene oxide [100] in an alkaline condition. This is because PCE’s extended side-chain structure provides the steric impedance necessary to maintain a satisfactory dispersion of the nanomaterials in pore solutions [99,100]. Similarly, it can aid in the efficient dispersal of NS in an alkaline condition [101]. Liu et al. [102] evaluated the scattering of NS in saturated Ca(OH)_2_ solution with and without the addition of PCE. They discovered that the mean diameter of NS treated with PCE (176.5 nm) was considerably smaller than the mean diameter of NS unmodified with PCE (7994.6 nm). Without PCE, NS will re-agglomerate in the pore solution, hence losing its unique nano characteristics, whereas PCE improves NS dispersion. Additionally, their findings indicated that NS treated with PCE might be employed to lower the porosity and quantity of hazardous voids in hardened cement paste. Feng et al. [103] discovered similar findings. In addition, another study suggested that altering the dispersion of NS will maximize the rheological properties of cement paste [104]. In general, it is critical to provide the adequate dispersal of NS in an alkaline solution in order to maximize cementitious materials’ function.

### 4.3. Fresh State Properties of NS-Modified Concrete

**Figure 5:**
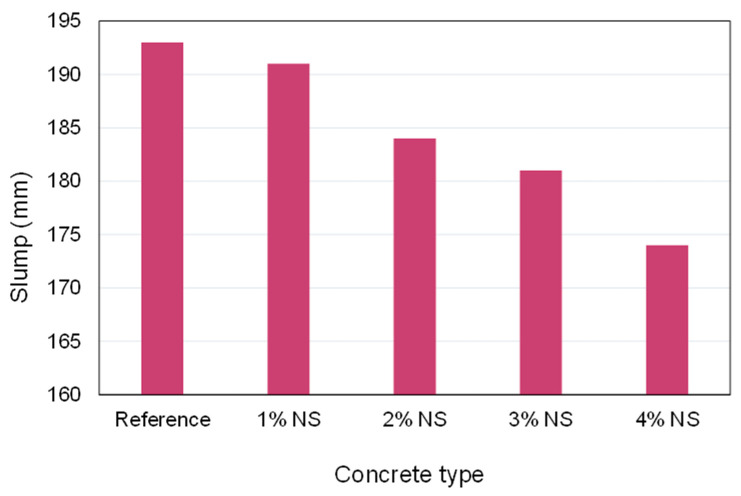
Influence of nano-silica dosage on the slump. Generated from the data obtained from [108].

The contact of NS in a fresh cementitious material mix demonstrates its effect on a variety of fresh mix properties, including consistency, setting time, and workability. Previous studies demonstrated that incorporating 2% NS into fly ash and slag cementitious materials sped up both the initial and final setting times [105,106]. Additionally, other studies observed a comparable impact of NS on the setting durations of ultra-high-performance concrete (UHPC) [107,108]. Numerous investigations have documented a decline in the workability of concrete using NS. The slump of fresh mix reduced as the percentage of NS increased from 1–4%, as shown in Figure 5. A rise in water demand was noticed as the proportion of NS in the cementitious materials increased, which might be attributed to the high SSA or fineness of NS grains, the rapid reaction of NS with the liquid face cement matrix, and the greater water absorption capability of NS [107]. Additionally, it was discovered that when the amount of NS in the concrete rose, the slump value was reduced [108]. Due to the large SSA and unsaturated bonds in NS, a part of the mixed water binds to the surface of the NS grains, forming silanol (Si-OH) groups. As a result, the amount of water required to maintain the workability of the fresh mix becomes inadequate. Ghafari et al. [107] found that the maximum quantity of NS that may be applied to maintain a suitable range of workability is 3% by weight, which was corroborated by [109]. The use of uniformly scattered NS improved the workability of the mix by approximately 35%, which might be attributed to the existence of free water within the ultra-fine NS grains, which enhanced the rolling effects among the grains [110]. This shows that a more uniform distribution of NP may possibly increase the workability of concrete. According to experiments on recycled aggregate concrete incorporating NS, the recycled aggregates and NS absorbed water, which resulted in slump loss, which was larger at higher NS dosages [111]. Elrahman et al. [112] showed that introducing NS lowered the consistency of the new mixture, hence increasing its viscosity. The air content of NS-incorporated concrete mixes was much higher than that of the control mix, owing to the greater viscosity of the paste containing NP with a large SSA [113]. By substituting NS for cement in self-compacting concrete (SCC) containing lightweight aggregates, the fresh density and consistency of the concrete were enhanced [114]. Cho et al. [115] observed that the addition of NS in small concentrations enhanced the rheological behavior of lightweight foam concrete (LWFC), as the stress growth rheometer test revealed a considerable rise in dynamic and static yield stresses.

### 4.4. Hardened State Properties of NS-Modified Concrete

#### 4.4.1. Compressive Strength (CS)

**Figure 6:**
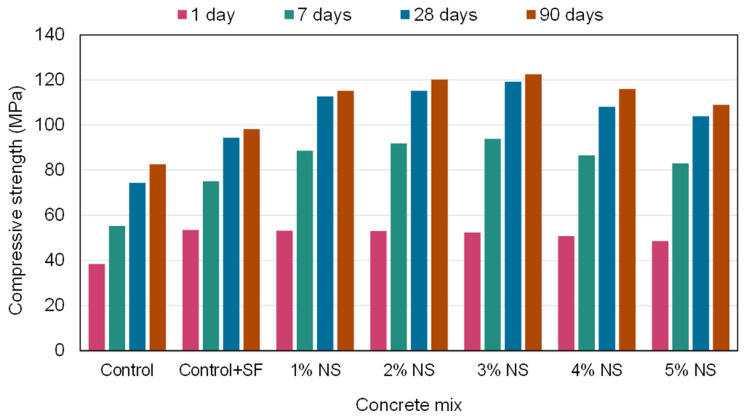
Influence of nano-silica dosage on compressive strength at different curing ages [131].

The incorporation of NS into concrete improves its CS. The CS of concrete modified with NS improves as the NS concentration approaches the threshold value. Increased NS content results in a drop in CS over the threshold value. CS is enhanced in NS concrete with an NS composition of 1.5%. When compared to normal concrete, the CS of NS-modified concrete improved by 16–25% after 7 days and by 12–17% after 28 days. The primary explanation for the increase in CS of concrete is the pozzolanic interaction between NS and Ca(OH)_2_, which results in the creation of C-S-H. However, without NS, concrete can only hydrate to a trace quantity of C-S-H by the action of cement. C-S-H is a critical component of strength. Hence, cementitious materials lacking NS have poor CS [116,117]. It was discovered that the influence of NS-modified cementitious materials on early strength is more pronounced, owing to the increased pozzolan reactivity of NS [44,118,119,120,121]. However, as the curing period increased, the quantity of NS particles employed in the pozzolanic reaction reduced, reducing the influence of NS-modified concrete on later-stage compression [122,123,124,125,126,127]. Ibrahim et al. [128] investigated the CS of NS-modified concrete that had been heated to a high temperature. The testing findings indicated that the samples containing NS had a greater effect on CS improvement at 400 °C. This might be due to the fact that as the temperature hit 400 °C, C-S-H with higher density was formed in the cementitious matrix, increasing the reactivity of NS, which accelerates the process of hydration and enhances the CS of concrete. The CS of concrete mixes at ages 1, 7, 28, and 90 days with increasing NS ratios is shown in Figure 6. As can be seen, NS enhanced the CS of concrete from 28 to 90 days, and the optimum quantity of NS is 3% by cement weight. CS improved by roughly 21% when compared to control mixtures [129]. The increase might be because Ca(OH)_2_ molecules in lime solution react with NS particles to generate extra C-S-H gel, hence enhancing the CS [125].

**Figure 7:**
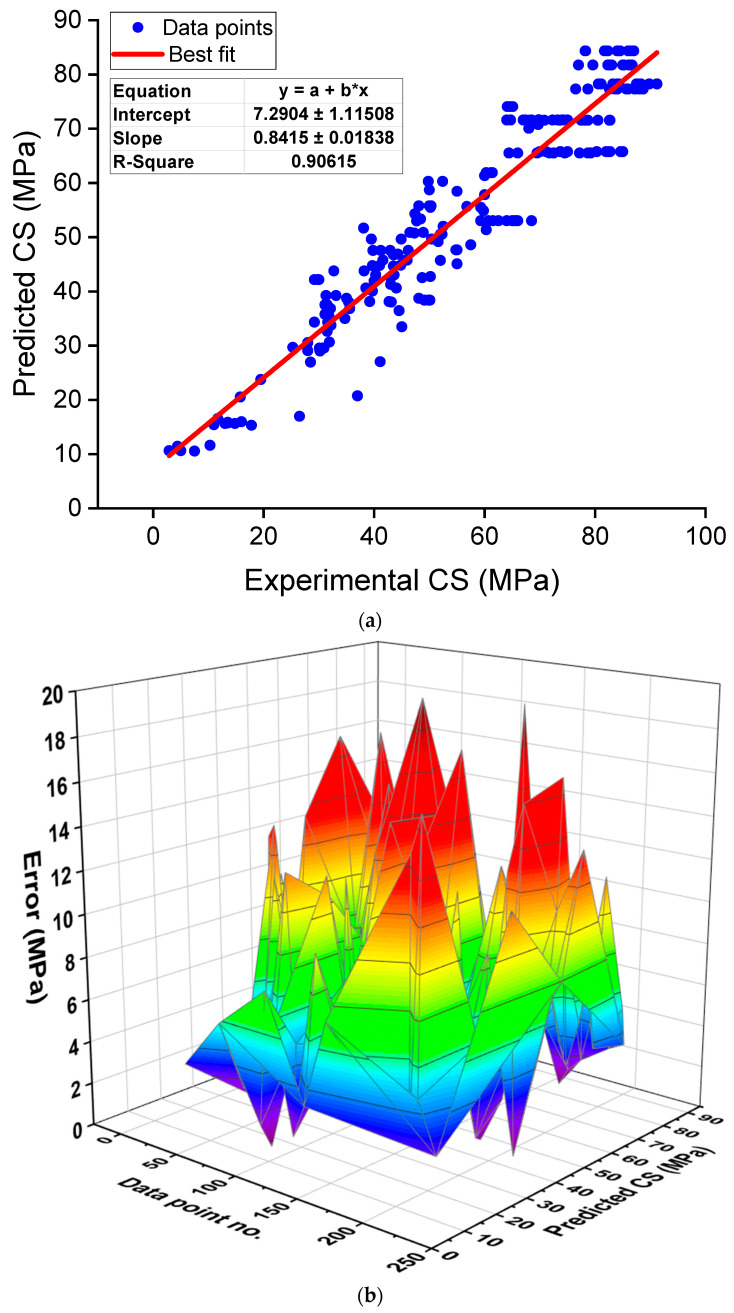
Regression model for CS: (**a**) correlation between actual and predicted results; (**b**) distribution of predicted and error values.

In addition to the discussion on the impact of NS on the CS of cementitious materials, this study performed a regression analysis of the experimental data retrieved from the literature using three essential variables, i.e., NS content, water-to-binder ratio (w/b), and the age of the specimen. A total of 218 data samples were collected from the relevant literature [105,111,117,130,131,132,133,134], and the correlation of NS content, w/b, and specimen age with CS was determined by developing a regression model, as shown in Equation (1). This equation can be employed to calculate the CS of NS-modified concrete for different NS contents, w/b, and specimen age. The relationship between experimental and predicted results is shown in Figure 7a. The coefficient of determination (R^2^) for an equation shows the accuracy of the model in predicting the results. A higher R^2^ value near 1 suggests a higher accuracy [135]. The resultant relation has an R^2^ of 0.91, which indicates a good agreement between experimental and predicted results. Moreover, the divergence of the estimated outcomes (error) from those of the experimental outcomes was analyzed and is displayed in Figure 7b. It was determined that the error values ranged between 0.01 to 19.22 MPa, with an average of 5.63 MPa. Additionally, of the 218 data samples, for around 22 samples, the error was less than 1 MPa, for 57 samples, the error was between 1 to 3 MPa, for 51 samples, the error was between 3 to 6 MPa, for 51 samples, the error was between 6 to 10 MPa, and the error was greater than 10 MPa for 37 samples. These error distributions also indicted a satisfactory performance of the regression for CS prediction of NS-modified concrete. 

However, to develop more accurate models, further experimental research needs to be conducted to collect more data samples. It is anticipated that using additional data samples would improve the predictive accuracy of the models.

(1) CS=−42.52+11.04 NS+494.73 wb+0.174 A−0.80 (NS)2−797.23 (wb)2+0.0003 A2

$$R^{2}=0.91$$

where

CS = predicted compressive strength,
NS = nano-silica content in percentage by weight,
wb = water-to-binder ratio of the mix, and
A = age of specimen in days.

#### 4.4.2. Split-Tensile Strength (STS)

**Figure 8:**
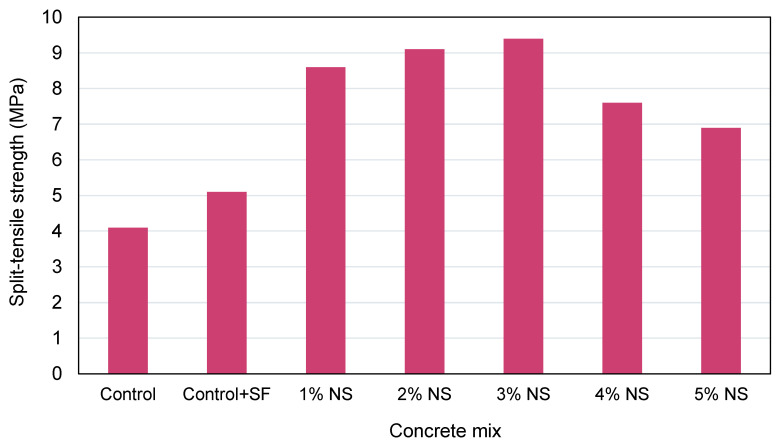
Influence of nano-silica dosage on split-tensile strength [131].

As anticipated, NS can enhance concrete’s STS. It was determined that the effect of 12 nm NS on the STS of concrete was greater than that of 7 nm NS since NS with a smaller SSA disperses more readily in water [119]. The STS of NS concrete was investigated by Fallah et al. [132]. When 3% NS was substituted for cement, the STS of NS-modified cementitious materials was increased by 16.1% compared to normal concrete. In comparison to the addition of NS to concrete, silica fume had a stronger strengthening impact on the STS of the concrete. The STS of cementitious materials incorporating 4% NS was enhanced by 35% when compared to normal concrete. As 4% NS was combined with 0.2% glass fiber, 0.2% polypropylene fiber, and 0.3% steel fiber, the STS of fiber-reinforced composites containing NS was enhanced by 77%, 57%, and 90%, respectively, when compared to the reference sample. This process occurs because NS strengthens the contact between the cement paste, fibers, and aggregates [136]. Not only does adding NS to concrete work as a nano-reinforcement, but it also performs as a filler, packing the voids in the cementitious mix [137,138]. Figure 8 shows the results of an experimental study, which indicated higher STS with NS addition [129]. The highest STS was noted with 3% NS content, while further addition of NS caused a reduction in the STS. This reduction in strength could be because the amount of NP in the mix exceeded the amount essential to react with the liberated lime during the process of cement hydration, resulting in excess silica leaching out and resulting in a strength decrease since it substituted part of the cement but did not contribute to strength. In addition, it is probable that weak regions were produced by flaws formed during nano-particle dispersal. The increased STS in NS-containing cementitious materials is because of the quick utilization of Ca(OH)_2_ generated during the hydration of cement, mostly at initial ages due to the strong reactivity of NS [129].

**Figure 9:**
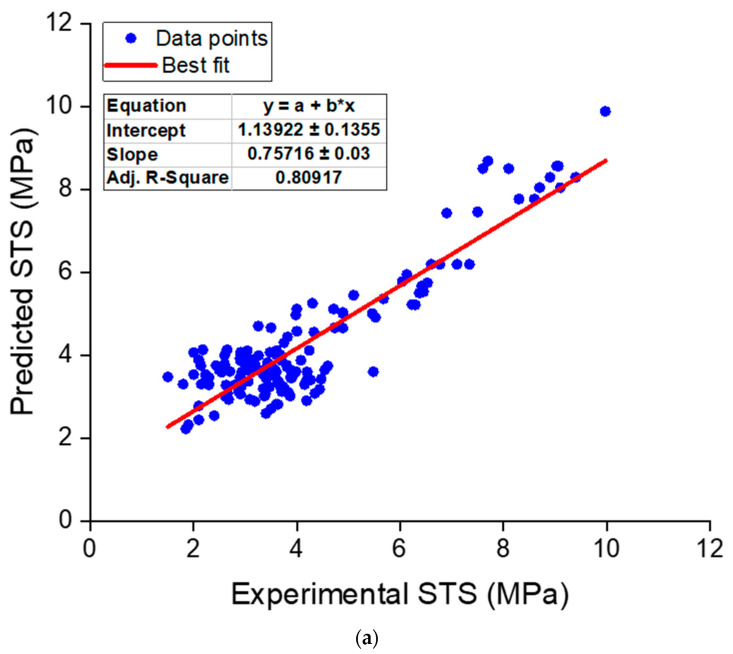

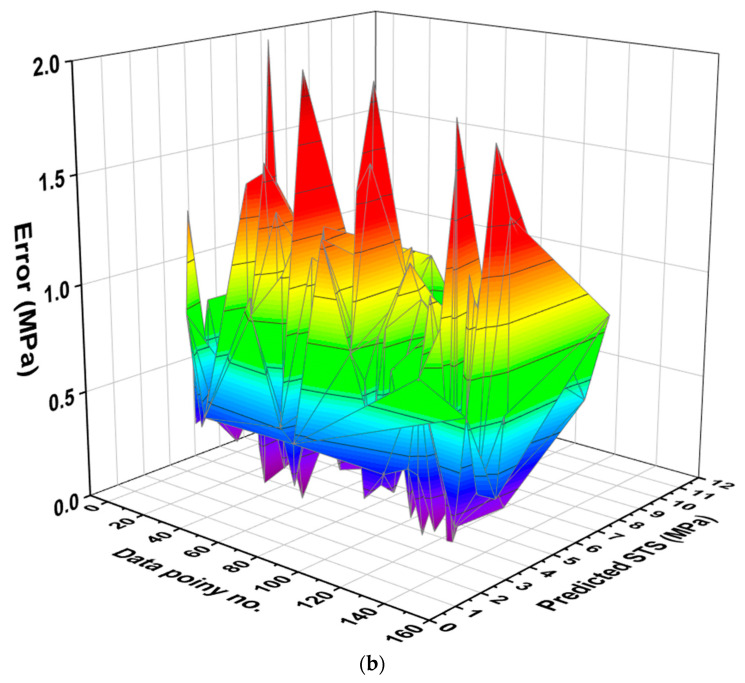
Regression model for STS: (**a**) correlation between actual and predicted results; (**b**) distribution of predicted and error values.

Similar to the CS, a regression analysis was also carried out for the STS data. A total of 151 data samples were collected from the relevant literature [109,111,117,129,132,139,140,141,142], and the correlation of NS content, w/b, and specimen age with STS was established by developing a regression model, as shown in Equation (2). This equation may be used to determine the STS of NS-modified concrete for various NS concentrations, w/b, and specimen ages. Figure 9a depicts the link between experimental and projected findings. The resultant model had an R^2^ of 0.81, indicating that experimental and anticipated findings corresponded well. In addition, the deviation (error) between the estimated and experimental outcomes was examined and is presented in Figure 9b. The error values were determined to vary from 0.002 to 2.07 MPa, with an average of 0.67 MPa. In addition, for about 65 of the 151 data samples, the error was less than 0.5 MPa, for 52 samples, the error ranged from 0.5 to 1 MPa, and for 34 samples, the error was larger than 1 MPa. These error distributions also indicated that the regression for STS prediction of NS-modified concrete performed satisfactorily. A lower accuracy of the STS model in comparison with the CS model is because of the fewer data samples employed since the experimental studies on the STS of the NS-modified concrete were less than the CS.

(2) STS=20.91+0.325 NS−72.37 wb−0.012 A−0.016 (NS)2+72.48 (wb)2+0.0001 A2

$$R^{2}=0.81$$

#### 4.4.3. Flexural Strength (FS)

**Figure 10:**
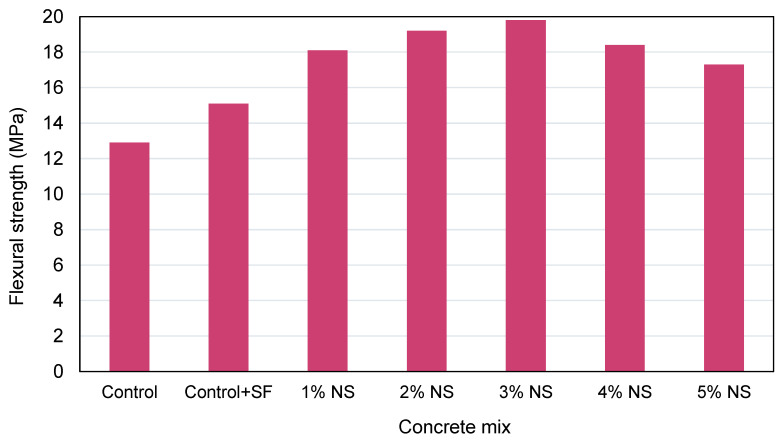
Influence of nano-silica dosage on flexural strength [129].

FS of cementitious materials containing NS was found to follow a comparable pattern to CS. Due to the changes in w/c, the ideal NS concentration varies, resulting in varying impacts on enhancing the FS of concrete [117,143,144,145,146,147]. Ltifi et al. [148] discovered that increasing the NS content of the mortar from 3 to 10% enhanced the FS. Rong et al. [116] discovered that, at an NS concentration of 3%, the NS-modified mortar had the maximum FS after 3 days, 7 days, 28 days, and 90 days of curing. According to Li et al. [149], the ideal FS content of UHPC is 1%. Wu et al. [150] investigated the FS of NS-modified fiber-reinforced concrete (FRC) at 25 °C, 375 °C, 575 °C, and 775 °C. It was discovered that FRC composed of 1% NS by weight and 0.15% carbon fiber by volume exhibited the maximum FS at room temperature and that the residual FS of FRC composed of various NS contents was improved to a greater or lesser level than that of FRC composed of 0.15% fiber at 375 °C, 575 °C, and 775 °C. This revealed that NS has a considerable influence on the flexural characteristics of FRC subjected to elevated temperature conditions. Beigi et al. [136] determined that a 4% NS concentration was best. The FS of cementitious materials containing 4% carbon fiber was raised by 40% when compared to conventional concrete. As NS was combined with various fibers (0.2% glass fiber, 0.2% polypropylene fiber, and 0.3% steel fiber), the FS of NS-modified FRC was enhanced by 75%, 53%, and 67%, respectively, when compared to the reference sample. This is primarily due to the NS pozzolanic and filler effect, which enhanced the fiber’s structural characteristics and adherence to the interface area. The results of experimental research are shown in Figure 10 [129]. Similar to the STS, the specimens’ FS improved with NS replacement up to 3% and subsequently fell, but the values at 4% replacement remained greater than those of the control concrete. Again, the increase in FS was attributed to the quick consumption of Ca(OH)_2_ generated during the hydration of cement, mainly at young ages, due to the strong reactivity of NS [129].

**Figure 11:**
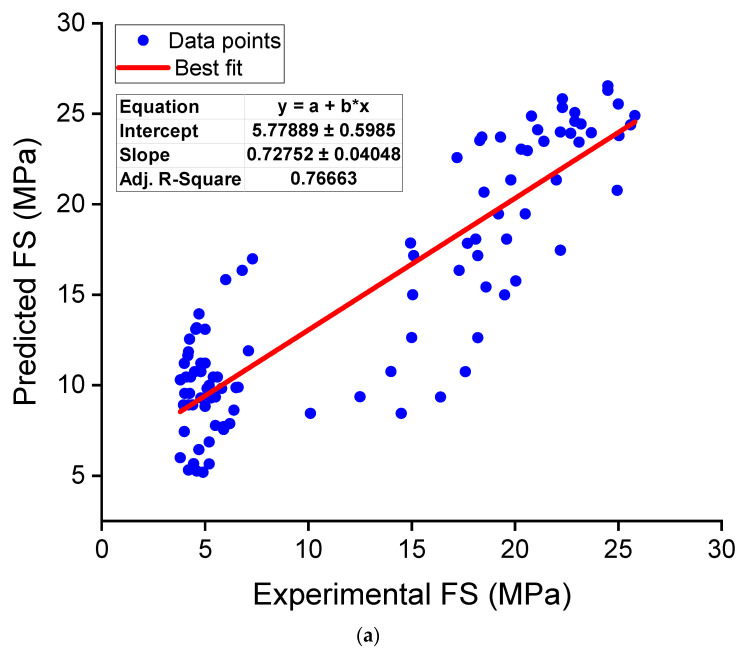

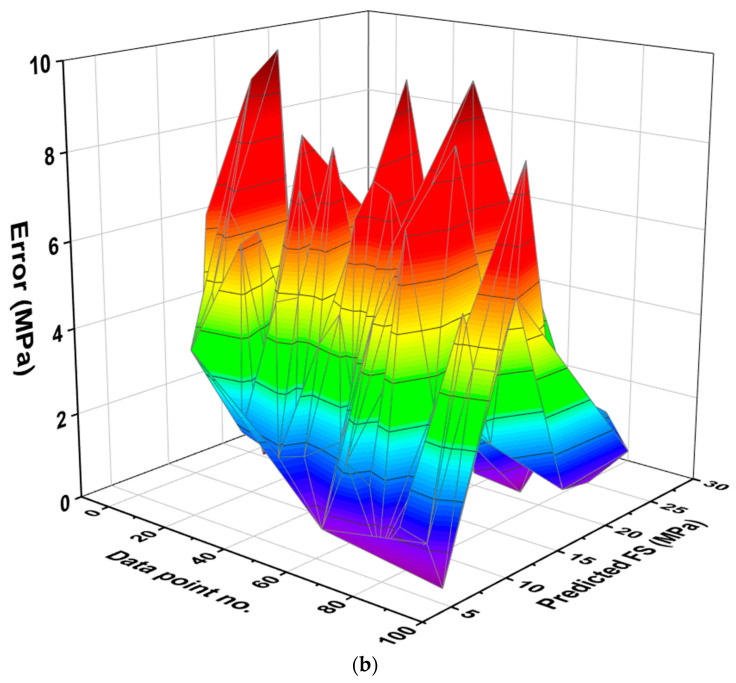
Regression model for FS: (**a**) correlation between actual and predicted results; (**b**) distribution of predicted and error values.

A regression analysis was also performed for the FS of NS-modified concrete. For this analysis, a total of 99 data samples were collected from the relevant literature [113,119,131,136,141,142,151]. The correlation of NS content, w/b, and specimen age with FS was determined by designing a regression model, as given in Equation (3). This equation may be used to calculate the FS of NS-modified concrete for different NS concentrations, w/b, and specimen ages. Figure 11a illustrates the relationship between experimental and predicted results. The resulting model had an R^2^ value of 0.77, suggesting that the experimental and predicted results did not agree well. This lower precision of the FS regression model might be due to the lower number of data samples employed compared to the CS and STS. In addition, Figure 11b displays the variance (error) between the estimated and experimental results. The error values ranged from 0.027 to 9.84 MPa, with an average of 3.69 MPa. Moreover, the error was less than 1 MPa for about 13 of the 99 data samples, between 1 and 3 MPa for 31 samples, between 3 and 5 MPa for nearly 27 samples, and greater than 5 MPa for 28 samples. These error distributions also revealed that the FS prediction regression for NS-modified concrete functioned less accurately. Hence, further studies are required to increase the number of data samples to develop accurate prediction models for the FS estimation of NS-modified concrete.

(3) FS=42.62+0.665 NS−141.61 wb−0.0127 A+0.243 (NS)2+140.68 (wb)2+0.001 A2

$$R^{2}=0.77$$

### 4.5. Durability Properties of NS-Modified Concrete

#### 4.5.1. Chloride Ion Penetration Resistance

Mercury intrusion porosimetry (MIP) studies indicated that the pozzolanic and filler properties of NS significantly inhibited the rate of chloride ion and water penetration at a low dosage of 0.3% NS [151]. It was reported by Isfahani et al. [152] that the chloride diffusion coefficient decreased at 0.5% NS dosages with water–binder ratios of 0.65 and 0.55; however, no such reduction was seen at higher NS dosages. The decrease in charge transmitted through the slag concrete was shown to correlate with the decrease in a critical maximum width of voids and improved microstructure caused by the addition of NS [105]. While lightweight concrete had a larger porosity of 2% NS, the increased binding capacity of C-S-H gel produced disrupted chloride ion transit [153]. Reduced conductivity in concrete caused less chloride ion diffusion into the matrix when a small quantity of NS was added [95]. The resistance of the NS concrete to chloride ion penetration was comparatively strong in comparison to the micro-silica and reference concretes [154]. 

#### 4.5.2. Carbonation

According to a study [84], the addition of 3% NS to fly ash–cement composites considerably decreased the carbonation depth by 73% after 180 days of exposure, compared to the control mix, but fly ash concrete containing silica fume exhibited a decrease of just 35% under a similar exposure situation. This is because the hydration products formed due to NS are more established and resilient to aggressive ion infiltration, resulting in a longer lifespan for fly ash concrete. Kumar et al. [155] showed that up to 3% NS lowered the depth of carbonation by 46% and 17% after 7 and 70 days, respectively, as compared to conventional concrete; however, increasing the NS dosage raised the depth of carbonation with time. The authors speculated that this is because excess Ca(OH)_2_ interacted with NS to create C-S-H gel, and a 3% dosage of NS resulted in a compact matrix, with a higher NS dosage having no effect on the density of the concrete. On the other hand, Isfahani et al. [152] found that the use of NS in cementitious materials had a negative impact on carbonation. The carbonation coefficient increased from 24 to 29.3 mm/year at 1% NS in a 0.65 water–binder ratio; the carbonation coefficient decreased from 20.4 to 16 mm/year in a 0.55 water–binder ratio with the same amount of NS. Additionally, Behfarnia et al. [124] described that the addition of NS particles to alkali-activated slag concrete increased the carbonation depth due to greater CO_2_ penetration.

#### 4.5.3. Water Absorption

Numerous investigations have demonstrated that NS can inhibit the adsorption of water and capillary absorption of cementitious materials. It was found that, after 28 and 90 days of curing, the rate and waste absorption coefficient of mix incorporating NS was considerably decreased [156]. Water absorption dropped from 5.60 to 4.41%, whereas the water absorption coefficient decreased from 2.86 to 1.39 (m^2^/s). Another study [157] examined the durability of cementitious materials containing between 0.3% and 0.9% NS. The authors discovered that utilizing a modest dose of NS is more successful at reducing permeability than using a high dose of NS since the NS can be effectively disseminated. Moreover, it was proven that permeability varied with NS particle size, i.e., the larger the particle size, the lower the permeability of the cementitious material [158]. This might be because the microstructure of concrete was enhanced using NP. Due to the influence of the nanofiller and the pozzolanic reactivity, the microstructure of cementitious composites became more homogenous and less porous, particularly at the ITZ, resulting in decreased permeability. In addition, hazardous substances’ pathways across the cement matrix might be partly filled and prevented [151].

#### 4.5.4. Freeze–Thaw Resistance

Not only may NS help prevent chloride ion and water penetration, but it can also help cementitious materials withstand freezing and thawing. It was found by Gonzalez et al. [159] that specimens with NS lost much less weight during freeze–thaw cycles. After 324 cycles, the mass of the control sample fell by 0.15%, whereas the mass of the sample containing 2% NS decreased by just 0.08%. The resistance to freeze–thaw is dependent on the mechanical properties, air void content, porosity, and other characteristics such as the distribution of air voids and the size of pores [113]. Therefore, it is possible to obtain a superior strength with a superior pore structure when utilizing NS in cementitious materials, causing greater resistance to water permeability and saturation [101].

#### 4.5.5. Shrinkage

Shrinkage develops in nearly every cement-based material as a result of the overall mass contracting owing to moisture loss through the components. The most often described influence of NS is its effect on the composite’s durability. Although NS has been shown to have therapeutic benefits, its proportion needs to be monitored. Shrinkage of the cement-based material is the primary cause of fractures that compromise the durability of the structure. Robertson [160] discovered that incorporating NS into cement-based materials incorporating pozzolanic materials can significantly minimize the autogenous shrinkage when compared to the reference mix. However, regular mortars incorporating NS shrunk more than the control mortar when dried. This effect was substantially more pronounced in the presence of a greater amount of NS. It can be avoided by using a small amount of super-plasticizer and following proper curing procedures [161,162]. Autogenous shrinkage owing to self-desiccation rises with increasing NS concentrations, leading to a larger cracking potential [163]. To avoid this negative impact, large concentrations of super-plasticizer and water should be used in conjunction with favorable curing conditions [164].

#### 4.5.6. Sulphate Resistance

**Figure 12:**
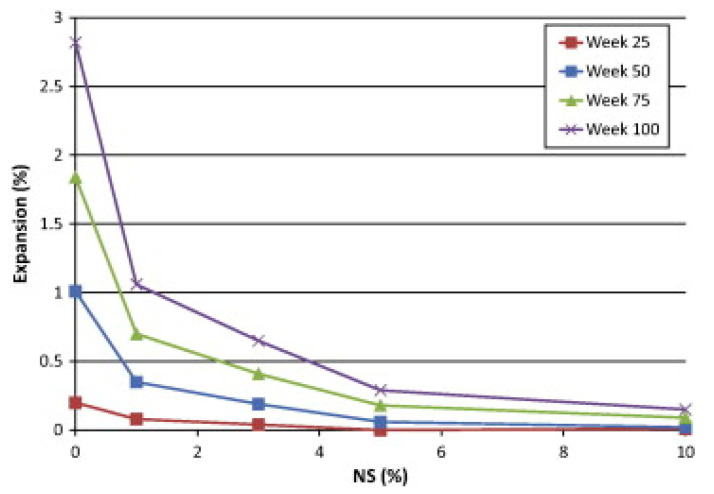
Influence of nano-silica addition on sulphate resistance of cementitious materials [169].

Sulfate resistance of concrete refers to the material’s capacity to withstand the development of secondary ettringite because of the subsequent reactivity of sulphate ions with hydrated aluminate phases. The development of secondary ettringite is an expanding reaction that may wreak havoc on the concrete matrix. Permeability of concrete is critical for sulphate resistance. As a result of the NS grains’ capacity to act as an inner filler inside the cementitious mix, the NS may be utilized to lower the permeability of the concrete by refining the pore structure. Arel and Thomas [165] studied the efficacy of NS and micro-silica as a partial substitute for cement in the presence of sulphate assaults. Their mortar mixes had a w/c of 0.5 and were subjected to a 7% sodium sulphate solution for 23 weeks. They determined that NS with an 8% substitute rate is sufficient to produce concrete that is extremely resistant to external sulphate assaults. Moreover, the authors indicated that the benefit of NS materials over micro-silica materials is their capacity to increase physical adhesion at the aggregate–cement interface, resulting in an excessive decrease in overall porosity and, hence, a reduction in chemical species’ permeability into concrete. Moslemi et al. [166] reached a similar outcome in their assessment of mortar resistance to sulphate assault. Their findings demonstrated that substituting 8% NS for cement would be adequate to increase concrete’s sulphate resistance. Tobón et al. [167] found that adding 3% NS can result in up to a 63% reduction in expansion when compared to a control specimen, showing that a lower replacement amount of NS may be useful for increasing concrete resistance to sulphate. Figure 12 is based on the work of Tobón et al. [167] and depicts the expansion of mortar subjected to a 5% MgSO_4_ solution in this investigation. This was also corroborated by research performed by Atahan and Dikme [168], who concluded that even a 2% substitution of NS for cement would be sufficient to successfully prevent expansion caused by sulphate attack. They both agreed that NS contributes to the refinement of the concrete pore system by increasing the smaller holes (gel pores) and decreasing the linked pores (capillary pores), thereby greatly enhancing the concrete’s durability. Thus, aggressive MgSO_4_ agents are unable to enter the cementitious matrix and generate expansive products.

#### 4.5.7. Acid Resistance

The CS of the NS-containing sample subjected to H_2_SO_4_ rain was much greater than the reference specimen. This is because the filling action of NS decreases the porosity of the sample and enhances the bonding between the cement paste and aggregates due to the addition of NS [169]. Additionally, the study discovered that as the NS concentration increased, the proportion of weight loss of the concrete specimens was reduced. Compared to the usage of NS, silica fume in high-strength FRC improved the durability of samples subjected to an acid condition [170]. After 63 days of immersion in H_2_SO_4_, the high-strength FRC samples containing 2% NS displayed a significant reduction in ultrasonic pulse velocity (UPV) in comparison with the 10% silica fume-containing samples. Moreover, the previous study demonstrated that the specimens lost less weight, suggesting less erosion during the acid exposure when a high substitution amount of 10% silica fume was used rather than a low substitution amount of 2% NS. This is because of the lesser possibility for the formation of gypsum and ettringite in cement-based materials incorporating 10% silica fume. In addition, the higher the silica fume substitution amount was compared with the lower NS substitution amount resulted in a larger crushing load loss-to-weight loss ratio.

### 4.6. Microstructure of NS-Modified Concrete

Based on the chemical characteristics of NS, it has been shown that the NS reacted with free lime (Ca(OH)_2_) during the hydration processes. As a result of the great fineness of the NS particles, this procedure resulted in the creation of C-S-H gel [171]. The size of NS grains is around 1–100 nm, which is favorable for reacting as a nucleus. As a result, thick cement hydrate products will be produced with an enhanced ITZ, even at low replacement levels [172]. The permeability of concrete can be used to determine its durability. The presence of NS affects the chemical and physical characteristics of cementitious materials. The NS pozzolanic reactivity with Ca(OH)_2_ results in the formation of additional C-S-H gels. Physically, NS is about 100 times finer than cement grains, is capable of filling residual gaps in cement paste, and improves the density of the matrix during the early ages [163]. Microstructural investigations of concrete using several electronic microscopy methods (SEM, ESEM, and TEM) revealed that cementitious materials with NS have a more homogeneous and denser microstructure than concrete without NS [163,164]. Due to the interface, filler, and filler impacts of NP, using NS can improve the microstructural properties of concrete. The NS can function as an activator, enhancing concrete’s hydration processes and forming huge volumes of C-S-H gel [134,173,174,175]. Thus, the incorporation of NS into cement pastes lowers calcium leaching and enhances durability [176]. Said et al. [95] conducted thermal and microstructural experiments to determine the influence of NS on the filler and pozzolanic processes. Due to the nucleation action of NP, the concrete pore structure with NS was enhanced. Lin et al. [177] discovered that adding NS (1–2%) lowered the relative permeability and pore diameters of mixes using an MIP test.

**Figure 13:**
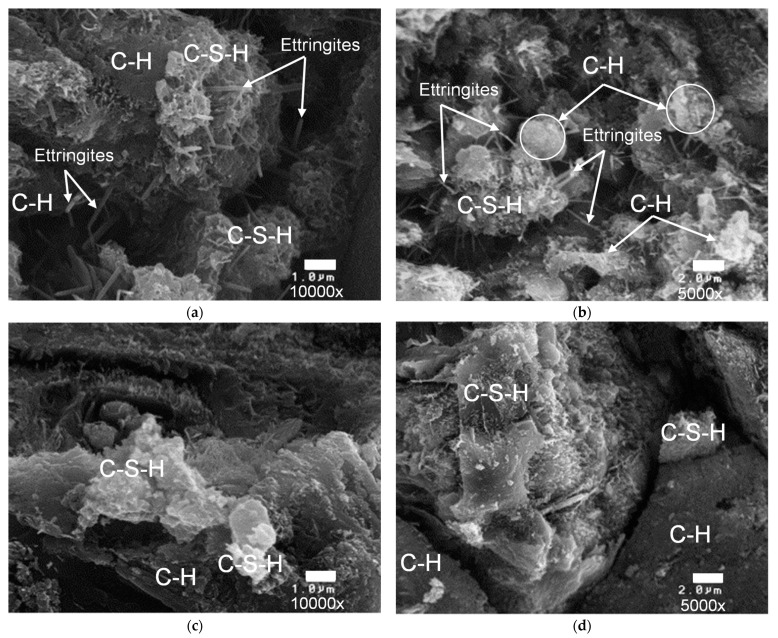
Microstructure of matrices: (**a**) cement paste 10,000×; (**b**) cement paste 5000×; (**c**) cement paste with NS 10,000×; (**d**) cement paste with NS 5000× [178]; CH: Ca(OH)_2_.

SEM studies were conducted by Jo et al. [178] to confirm the mechanism anticipated by the CS test. It was discovered that the incorporation of NS particles altered the hydration behavior and resulted in changes in the microstructure of the hardened pastes. SEM micrographs of cement paste with and without NS after 7 days are shown in Figure 13. In the SEM micrograph of the cement paste, the C-S-H gel was isolated, bounded by and linked to many needle hydrates called ettringites (Figure 13a,b). On the other hand, the microstructure of the cement paste containing NS demonstrated the production of dense, compact hydration products and a decreased quantity of Ca(OH)_2_ crystals (Figure 13c,d). These observations are in line with the enhanced mechanical and durability properties of NS-modified concrete.

### 4.7. Challenges to the Use of NS in Concrete

Along with the expensive cost, there are certain barriers to the large-scale application of NS. Between laboratory investigation and on-site application, there is a significant gap. The first issue to address is NS dispersal. It is challenging to have huge ultrasonic equipment or sufficient space on site to meet NS dispersal requirements. Moreover, the ultrasonic therapy technique is lengthy and difficult to regulate. Although a construction firm can procure NS dispersal directly from producers for on-site usage, the materials and transport expenses are substantially high, further increasing the expense of the NS application. Second, the toxicity of NS will result in pulmonary complications and an increase in mortality [179,180]. As a result, laborers must adopt stringent protective measures to maintain an adequate level of safety and health on the job. Thirdly, it is critical to monitor and manage NS dispersion on site in order to reduce the danger to the surrounding environment. Thus, environmental, economic, and health considerations should be addressed prior to the widespread use of NS.

### 4.8. Future Research Directions

Nanotechnology enables modification of the basic structure of materials in order to improve their characteristics. In concrete, NS works as a nucleation site, accelerating the cement hydration and filling the voids, resulting in a higher packing density and lower porosity. However, to fully exploit the advantages of nanotechnology, upcoming research should solve the following concerns.

- A significant concern is the physical condition and dispersion of NS in concrete. While numerous dispersion agents are in use, their practicality in the field remains debatable. A comprehensive examination of the dispersion process is necessary.
- The optimal amount of NS in cementitious materials cannot be quantified in terms of a percentage. It is entirely dependent on the kind of NS (powder/colloidal) and its average grain size, which may be described in terms of surface area-to-mass ratio. In this regard, a link between the optimal quantity and features of NS should be established.
- While most studies have been conducted on mortars and cement pastes, a limited number of scholars have concentrated on the mechanical characteristics and permeability of NS-modified concrete. Other durability characteristics remain less explored. Due to the integration of finer materials, shrinkage behavior may be changed, which should be investigated in depth. Additional research on acid, sulphate, carbonation, and chloride resistance is required.
- Even though scholars have explored some characteristics of cementitious materials, they are not sufficiently confident in developing for their application in construction. A comprehensive, systematic experimental investigation needs to be conducted to determine the properties of NS-modified concrete. The optimization of concrete’s fresh, mechanical, microstructural, and durability characteristics must be pursued vigorously.
- ITZ research is an exciting field in which to discover new things. Because ITZ exhibits behavior that is distinct from the other two phases of concrete, it is the chain’s weakest link and, as such, its nanomechanical characteristics should be determined.
- An extensive study into the mathematical modeling or machine learning of concrete behavior is still possible. A study of this area may yield fresh results that contribute to a better understanding of concrete. However, no considerable research has been conducted in these areas to date.

## 5. Conclusions

The intention of this study was to carry out a keywords’ evaluation of the published literature on nano-silica (NS)-modified concrete to analyze diverse crucial portions of the current research. The Scopus database was searched for 1015 relevant documents, and the data were evaluated using the VOSviewer software. In addition, this study comprehensively discussed the crucial segments in the present research area and developed prediction models for the strength estimation of NS-modified concrete. The following conclusions are drawn from this study:

- The analysis of keywords revealed that the five most regularly appearing keywords are nano-silica, silica, compressive strength, concretes, and cements. The assessment of keywords showed that NS had been researched mainly to enhance the durability, mechanical, and microstructural characteristics of concretes
- The inclusion of nano-particles such as NS in cementitious materials necessitates the addition of more water or super-plasticizers to preserve the workability of the fresh mix.
- The impact of NS on cementitious materials is dependent on the particle size, kind (colloidal/powder), surface area, dose, and the w/c of the mix.
- Increasing the NS concentration up to 2% and 3% might enhance the mechanical and durability properties of cementitious materials. This might be because of the pozzolanic reactivity, the refinement of the pore structure, or the filling effect.
- The mechanical strength of concrete improves with increasing the NS concentration, and the NS acts as an activator, promoting the hydration process. However, if the concentration of NS is greater than 3%, the strength may be reduced.
- The prediction models established for the strength prediction of NS-modified concrete showed good agreement with the experimental data due to higher R^2^ and reduced error values. This sort of analysis might be used to estimate the required parameters of material, therefore saving time and money associated with experimental studies.
- An increased NS dose, i.e., 3% or more, may deteriorate the characteristics of the material because of the accumulation of NS grains, resulting in increased porosity, microcracking, and decreased mechanical strength.
- NS has a substantially stronger pozzolanic activity than silica fume. At all ages, concrete containing NS displayed a greater compressive strength than conventional concrete. Additionally, the incorporation of NS increased the concrete’s flexural and split-tensile strengths.
- As a consequence of the filler effect and pozzolanic activity of NS, as well as its interaction with Ca(OH)_2_ crystals to lower their size and quantity, a compact ITZ microstructure formed between the cement paste and aggregates, resulting in the long-term strength growth and durability of the matrix.
- If the NS particles are equally scattered, the inclusion of NS particles enhances the microstructure of the concrete.
- Even though NS exhibited noteworthy beneficial influences on the durability parameters and mechanical properties of the cementitious materials in a variety of conditions and environments, there remain divergent views on the size and kind of NS, its concentration, and dispersal techniques. Therefore, a broad study in this field is necessary to establish fundamental requirements for the practical deployment of such nano-particles.

## Figures and Tables

**Table 1:** List of top 20 frequently used keywords in the research of NS concrete.

S/N	Keyword	Occurrences
1	Nano-silica	539
2	Silica	533
3	Compressive strength	393
4	Concretes	343
5	Cements	202
6	Durability	192
7	Silica fume	159
8	Fly ash	154
9	Mechanical properties	152
10	Concrete	141
11	Scanning electron microscopy	137
12	Hydration	123
13	Nano-particles	108
14	Tensile strength	108
15	Microstructure	106
16	Mortar	103
17	Concrete mixtures	99
18	Aggregates	96
19	Water absorption	93
20	Portland cement	87

**Table 2:** Physical properties of nano-silica from the literature.

Reference	Type	SSA (m^2^/g)	Size (nm)
[86,87]	Powder	640	15–20
[88]	Colloidal	954.3	10
[89]	Powder	175–225	30–70
[90]	Powder	300	7–40
[91]	Powder	240	7–25
[92]	Powder	125	20–30
[93]	Powder	120–230	10–150
[94]	Colloidal	-	10–140
[95]	Colloidal	-	35

**Table 3:** Chemical composition of nano-silica.

Chemical Compound	Composition (%)			
Reference	[96]	[86]	[91]	[97]
SiO_2_	95.00	99.50	90.90	99.65
Al_2_O_3_	1.08	0.002	0.29	0.01
Fe_2_O_3_	0.45	0.001	0.10	0.012
MgO	1.06	0.002	0.15	<0.01
CaO	0.20	-	0.19	<0.01
SO_3_	0.31	-	1.16	<0.01
K_2_O	0.12	-	-	<0.01
Na_2_O	0.68	-	1.1	<0.01
TiO_2_	0.18	-	0.29	0.02
P_2_O_5_	0.12	-	-	<0.01

## Data Availability

The data used in this research has been properly cited and reported in the main text.
